# Crossover Analysis Is Needed in Smartphone-Supported Vestibular Rehabilitation

**DOI:** 10.2196/107851

**Published:** 2026-09-30

**Authors:** Jin Yan, Kaiyuan Yang, Xixin Wang, Boyan Su

**Affiliations:** 1 Clinical Cognitive Neuroscience, Advanced Occupational Therapy, Human Health Sciences, Graduate School of Medicine, Kyoto University, Kyoto, Kyoto, Japan; 2 Department of Neurosurgery, Beijing Tsinghua Changgung Hospital, School of Clinical Medicine, Tsinghua Medicine, Tsinghua University, 168 Litang Road, Beijing, 102218, China, 86 01056119530, 86 01056119530

**Keywords:** vestibular rehabilitation, smartphone, crossover trial, digital health, statistical analysis

Kaplan et al [1] evaluated smartphone-supported vestibular rehabilitation in a randomized 2-period, 2-sequence crossover pilot trial and interpreted their findings as suggesting app support conferred an advantage. However, the reported analyses did not directly estimate the incremental effect of the app.

Participants were randomized to app-first or conventional-first sequences, crossed over after 3 weeks, and had no washout period; residual effects may therefore have influenced period 2 [1]. The CONSORT (Consolidated Standards of Reporting Trials) crossover extension emphasizes paired participant-level analyses that account for within-participant correlation [2]. In contrast, Mann-Whitney *U* tests were used to compare sequences, whereas Friedman and Wilcoxon tests were used to compare time points within sequences. A comparison between baseline and week 6 provides an estimate of the total 6-week change, and a between-sequence comparison of that change provides an estimate of the order difference after both sequences received both delivery modes. Neither isolates app support from conventional delivery.

The Instrumented Timed Up and Go Test result illustrates the problem. In the app-first sequence, the only significant post hoc change was from baseline to week 6, spanning both treatment periods, not from baseline to week 3, the app-supported period. The results therefore cannot establish an early benefit of smartphone guidance. Moreover, significance in one sequence but not the other does not establish a difference between treatments or sequences [3].

The Dizziness Handicap Inventory responder analyses have the same limitation. At week 6, 7 of 9 eligible app-first participants and 2 of 8 conventional-first participants exceeded the minimal clinically important difference, but both sequences had received both modes [1]. The period-specific counts (6/17 vs 3/17) are paired outcomes, so discordant-pair counts are needed. Two *P* values also require clarification. For the week 6 table, 7 of 9 versus 2 of 8 yields a likelihood-ratio *χ*^2^_1_=4.98 (*P*=.026), whereas the 2-sided Fisher exact test gives *P*=.057; the manuscript reports *χ*^2^_1_=4.97 (*P*=.06). For period-specific responders, treating 6 of 17 and 3 of 17 as independent observations yields *P*=.438 with the 2-sided Fisher exact test, not *P*=.057. However, because these are paired outcomes, the appropriate analysis requires the discordant-pair counts, which were not reported.

We recommend reporting period-specific outcomes using a participant-level model that estimates treatment and period effects and considers sequence and carryover. Given the absence of washout and plausible persistence of rehabilitation effects, an exploratory baseline-adjusted first-period comparison preserving randomization may be more interpretable than a full crossover contrast. Until then, the study supports feasibility and overall improvement across 6 weeks, but does not establish an incremental benefit of app support.

## Abbreviations

CONSORT: Consolidated Standards of Reporting Trials

## References

[1] Kaplan A, Kalderon L, Wolfovitz A, Gimmon Y, Levy-Tzedek S (2026). Smartphone-supported vestibular rehabilitation in individuals with vestibular dysfunction: pilot randomized crossover trial assessing functional clinical outcomes and anxiety. JMIR Rehabil Assist Technol.

[2] Dwan K, Li T, Altman DG, Elbourne D (2019). CONSORT 2010 statement: extension to randomised crossover trials. BMJ.

[3] Gelman A, Stern H (2006). The difference between “significant” and “not significant” is not itself statistically significant. Am Stat.

